# Comparing the effects of the modified Social Cognition Individualized Activities Lab (mSoCIAL) between subjects with schizophrenia and those with borderline personality disorder: a pilot study

**DOI:** 10.1192/j.eurpsy.2026.11706

**Published:** 2026-07-31

**Authors:** P. Blasio, C. Blua, L. Giuliani, C. Recano, E. Chianese, P. Bozzatello, S. Bellino, A. Mucci, S. Galderisi

**Affiliations:** 1 Department of Psychiatry, University of Campania “Luigi Vanvitelli”, Naples; 2 Department of Neuroscience, University of Turin, Turin, Italy; 3 Department of Neuroscience, University of Turin, Turin, Italy

## Abstract

**Introduction:**

Social Cognition (SC) deficits significantly impact real-life functioning in individuals with schizophrenia (SCZ) and are emerging as relevant features also in subjects with borderline personality disorder (BPD). The modified Social Cognition Individualized Activities Lab (mSoCIAL) is a personalized rehabilitation program previously shown to improve SC and functioning in SCZ. However, no studies have assessed its transdiagnostic efficacy across different psychiatric populations.

**Objectives:**

This pilot study aimed to evaluate the effects of mSoCIAL on SC, neurocognitive abilities, narrative skills, and daily functioning in patients with BPD, and to explore the potential transdiagnostic applicability of mSoCIAL across in BPD and SCZ patients.

**Methods:**

Eight outpatients (4 SCZ, 4 BPD) underwent 10 weekly sessions of mSoCIAL. Participants were assessed before and after treatment using standardized tools for psychopathology, neurocognition, social cognition, functioning, and narrative coherence. Within and between-group differences were analyzed using paired-sample t-test and repeated-measures MANOVA

**Results:**

SCZ patients showed significant post-intervention improvements in narrative coherence for negative experiences and in communication skills, although these differences did not survive after correction for multiple tests. No significant changes were observed in BPD patients. However, both groups improved over time in emotion recognition, narrative coherence for negative episodes, and functioning domains, such as comprehension and daily activities. No significant time by group interaction emerged, suggesting comparable benefit across diagnoses.

**Conclusions:**

Despite the small sample size, findings suggest that mSoCIAL may enhance key domains of social cognition, cognitive abilities, narrative skills and certain domains of functioning in both patients with schizophrenia and those with BPD. These preliminary results support the potential transdiagnostic applicability of SC-focused interventions. Further research with larger samples is warranted to confirm these findings and explore the differential responsiveness based on baseline impairment levels.

**Disclosure of Interest:**

None Declared

